# Human Engineered Heart Tissue Models for Disease Modeling and Drug Discovery

**DOI:** 10.3389/fcell.2022.855763

**Published:** 2022-03-31

**Authors:** Hidenori Tani, Shugo Tohyama

**Affiliations:** ^1^ Department of Cardiology, Keio University School of Medicine, Tokyo, Japan; ^2^ Department of Emergency and Critical Care Medicine, Keio University School of Medicine, Tokyo, Japan

**Keywords:** engineered heart tissues (EHTs), human induced pluripotent stem cells (iPS cells) (hiPSCs), tissue engieering, disease model, drug discovery, cardiotoxicity

## Abstract

The emergence of human induced pluripotent stem cells (hiPSCs) and efficient differentiation of hiPSC-derived cardiomyocytes (hiPSC-CMs) induced from diseased donors have the potential to recapitulate the molecular and functional features of the human heart. Although the immaturity of hiPSC-CMs, including the structure, gene expression, conduct, ion channel density, and Ca^2+^ kinetics, is a major challenge, various attempts to promote maturation have been effective. Three-dimensional cardiac models using hiPSC-CMs have achieved these functional and morphological maturations, and disease models using patient-specific hiPSC-CMs have furthered our understanding of the underlying mechanisms and effective therapies for diseases. Aside from the mechanisms of diseases and drug responses, hiPSC-CMs also have the potential to evaluate the safety and efficacy of drugs in a human context before a candidate drug enters the market and many phases of clinical trials. In fact, novel drug testing paradigms have suggested that these cells can be used to better predict the proarrhythmic risk of candidate drugs. In this review, we overview the current strategies of human engineered heart tissue models with a focus on major cardiac diseases and discuss perspectives and future directions for the real application of hiPSC-CMs and human engineered heart tissue for disease modeling, drug development, clinical trials, and cardiotoxicity tests.

## Introduction

Cardiovascular disease remains a principal cause of death worldwide, and in an aging society, the number of patients with heart failure (HF) is increasing rapidly. In contrast, cardiovascular drug development is decreasing due to challenges in gauging the pathophysiology of many heart diseases and the effects of drugs on healthy hearts (Eder et al., 2016). This is not only because heart size, beat rate, and ion channel expression differ between humans and small animals, but also because variants and mutations that cause or predispose humans to cardiovascular diseases (CVDs) have an inconsistent impact on transgenic mice despite having a genetic equivalent (Greenberg et al., 2018). Moreover, drug development is lengthy and costly and is difficult due to the limited value of current preclinical assessment systems for predicting clinical adverse drug reactions, such as proarrhythmic and cardiotoxic effects. Thus, it is necessary to develop robust and reproducible heart models based on human cells for drug discovery.

Since the development of human induced pluripotent stem cells (hiPSCs) and stable differentiation of hiPSC-derived cardiomyocytes (hiPSC-CMs) has been achieved, many efforts have been made to fabricate heart models for use in disease modeling, drug efficacy or toxicity testing, and mechanistic studies. Such efforts have been accelerated by improvements in bioengineering and *in vitro* culture technologies. These fields can be developed by precise gene editing using CRISPR-Cas9 technology, such as generation of transgenic reporter cell lines and introduction and restoration of CVD-relevant mutations to create isogenic lines of diseased and healthy cells (Passier et al., 2016). There is now a wide spectrum of patient hPSC (human embryonic stem cell [hESC] and hiPSC) lines with genetic mutations for cardiomyopathies available (Giacomelli et al., 2017b). They can also be differentiated into hPSC-derived cardiomyocytes (hPSC-CMs) with functional abnormalities. Although such growing application provides an advantage of hPSC-CMs, a limitation of hPSC-CMs is their immaturity.

One of the most effective solutions to this shortcoming is the application of three-dimensional (3D) tissue engineering techniques using relevant sensors and external stimuli, such as mechanical and electrical stimulations, cardiac microtissues (CMTs), engineered myocardium, and heart-on-a-chip (HoC) devices (Eder, et al., 2016; Giacomelli et al., 2017a; Zhang et al., 2015). Notably, scaffold-based 3D cardiac models such as engineered heart tissues (EHTs) have shown that anisotropic mechanical limitation of cardiomyocyte (CM) contractility can predispose hPSC-CMs to functional and morphological maturation (Zimmermann et al., 2002). Furthermore, maturation is enhanced by exposure to cyclic stress and contact with non-CM cells in 3D cardiac models (Ronaldson-Bouchard et al., 2018).

In addition to determining the mechanisms of genetic or acquired diseases and drug responses, owing to mature 3D cardiac models, hPSC-CMs have been expected to detect arrhythmias, contractile dysfunction, and other abnormalities when applied in clinical trials and cardiotoxicity tests. Here, we provide an overview of the latest engineered models of hPSC-CMs and discuss their applications in human heart research.

## The Challenge of hiPSC-CMs

Various attempts to artificially develop differentiated CMs from hPSCs have been made, which have been accelerated since hiPSCs were developed by Yamanaka et al. (Takahashi et al., 2007). This technology generates personalized stem cells derived from healthy individuals and patients. Highly efficient differentiation protocols using low-molecular-weight compounds and metabolic selection methods have enabled researchers to obtain large quantities of purified hiPSC-CMs adequate for hPSC-CM transplantation therapy (Minami et al., 2012; Tohyama et al., 2013; Tohyama et al., 2016; Tanosaki et al., 2020; Someya et al., 2021; Tani et al., 2022). Because hiPSC-CMs are human-derived, readily available, and can be maintained *in vitro* for months, they have attracted increasing attention not only as a replacement for conventional heart transplantation but also for cardiomyopathy modeling, and they may provide new insights into the mechanisms of disease phenotypes. Novel gene therapy based on CRISPR/Cas9 and adeno-associated virus has also been advocated as a tool in combination with patient-specific disease models. Drug toxicity screening is another promising application of hiPSC-CMs. In fact, a new paradigm based on hiPSC-CMs has been proposed to more accurately predict proarrhythmic risk (Blinova et al., 2018).

**FIGURE 1:**
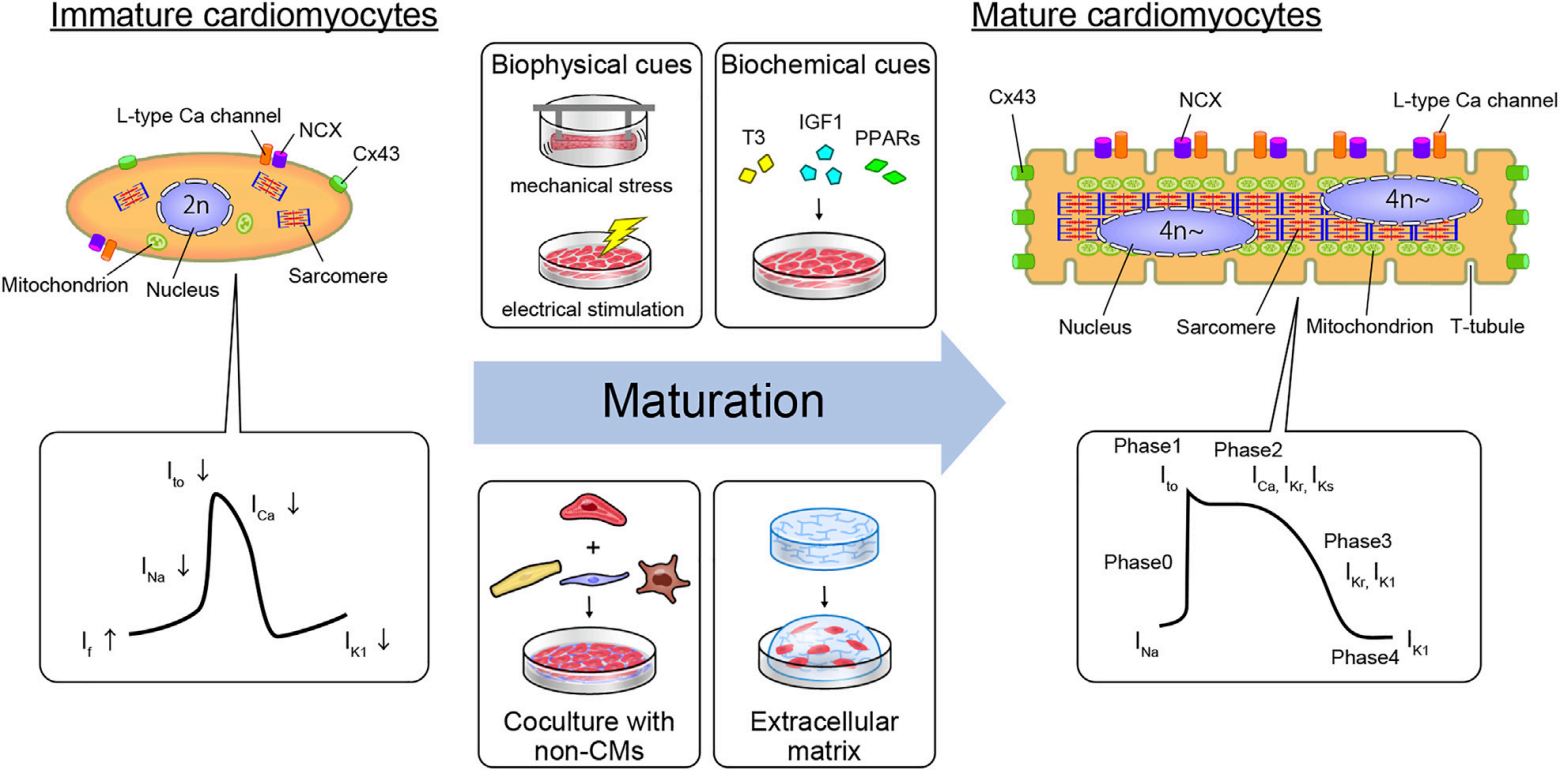
Cardiomyocyte maturation features and strategy to generate mature hiPSC-CMs *in vitro*. The structural and electrophysiological features of hPSC-derived CMs resemble those of fetal human CMs but not those of adult human CMs. Mature humans and immature CMs contain different features in their constituents. Moreover, APs differ due to differences in the expression levels of some ion channels. Immature hPSC-derived CMs can be driven toward a more mature adult CM phenotype using biophysical cues (e.g., mechanical stress or electrical stimulation), biochemical cues (the addition of T3, IGF1, or PPARs to the culture medium), coculture with non-myocyte cell types such as CF, EC, and macrophages, or by growing in the extracellular matrix. Abbreviations: CM, cardiomyocyte; CF, cardiac fibroblast; Cx43, connexin 43; EC, endothelial cell; IGF1, insulin-like growth factor 1; ICa, calcium channel current; If, pacemaker or funny current; IK1, inward-rectifier potassium current; IKr, rapid delayed-rectifier potassium current; IKs, slow delayed-rectifier potassium current; INa, sodium current; Ito, transient outward potassium current; NCX, sodium-calcium exchanger; PPARs, peroxisome proliferator-activated receptors; T3, triiodothyronine.

However, one of the most important hurdles in modeling cardiac disease or drug responses with hPSC-CMs is their immaturity, both structurally and functionally (Tani, et al., 2022) (Figure 1). Immaturity is a significant disadvantage when *in vitro* model systems are expected to resemble adult human CMs in form and function. The growth of the prenatal heart is primarily driven by the proliferation of CMs, a process called hyperplasia. During postnatal development, CMs quit the cell cycle and generally undergo one final round of DNA synthesis without cell division, generating large tetraploid nuclei (Mollova et al., 2013). CMs then grow largely, with a 10- to 20-fold increase in cell volume, resulting in hypertrophy of the muscle fibers. The profile of hPSC-CMs is known to be the same as that of prenatal cells with regard to structure, gene expression, energy, force, conduct, ion channel density, and Ca^2+^ kinetics. In contrast to human mature CMs, immature CMs do not have anisotropic, rod-like shaped, ordered myofibrils, contractile cytoskeletons, T-tubules, nuclei with abundant DNA, junctions, elevated calcium signaling, and other organelles (Figure 1). Notably, components such as mitochondria can mature morphologically in proportion to CM maturation. Mitochondria in immature CMs are circular and small in number and size and are situated around a nucleus with sparse cristae density, whereas mitochondria in mature CMs are oval and large in number and size, with thick cristae density. Moreover, such mature organelles are aligned in an orderly manner between myofibrils and under the sarcolemma (Hom et al., 2011).

From an electrophysiological perspective, hPSC-CMs are immature. Cardiac action potential (AP) is orchestrated by various ion channels and presents four clear phases in mature CMs. In phase 0, AP is initiated by rapid sodium influx (I_Na_) depolarization. Thereafter, a transient outward potassium current (I_to_) causes repolarization in phase 1. Phase 2 is a plateau phase mediated by L-type calcium channels (I_CaL_). During phase 3, the rapid delayed-rectifier potassium currents (I_Kr_) and slow delayed-rectifier potassium currents (I_Ks_) open, leading to repolarization. In contrast, immature CMs differ from mature CMs in various ways. First, the resting membrane potential (RMP) of immature CMs is less negative (approximately -60 mV, similar to that of nodal cells; mature CMs approximately -85 mV) as a result of insufficient expression of the inward-rectifier potassium current (I_K1_), which is mediated by the inward-rectifier potassium channel Kir2.1 (encoded by KCNJ2) (Goversen et al., 2018). Second, the upstroke velocity of immature CMs is slower than that of mature CMs (mature CMs max dV/dt of approximately 200 V/s), which is caused by the fetal isoform of the Nav1.5 α-subunit of the sodium channel (encoded by SCN5A) and fewer other sodium channels (Yu et al., 2011; Veerman et al., 2017). Third, the plateau phase of the AP is shorter in immature CMs, partly due to the lower expression of the Cav1.2 core component CACNA1C and alternative splicing of its auxiliary subunit CACNB2 (Qu and Boutjdir, 2001; Link et al., 2009). This lower Cav1.2 activation is related to inadequate Ca^2+^ handling, leading to immature excitation contraction coupling. Moreover, the fast repolarization phase in immature CMs is mostly mediated by I_Kr_ without I_Ks_ mediation (Hoekstra et al., 2012; Zhao et al., 2018). Thus, pharmacological inhibition of I_Kr_ leads to a significant increase in the AP duration (APD), which reduces the beat rate in immature hPSC-CMs (Doss et al., 2012). Whereas mature ventricular CMs exhibit low automaticity, immature CMs spontaneously beat. Multiple factors contribute to the automaticity of immature hPSC-CMs, including the expression of pacemaker channels such as HCN4 (hyperpolarization-activated cyclic nucleotide-gated potassium channel 4), a RMP that is closer to the AP activation threshold, and spontaneous Ca^2+^ release, which drives membrane depolarization through the Na^+^-Ca^2+^ exchanger (NCX) (Kim et al., 2015). Although all the mechanisms have not yet been elucidated, such differences cause inaccuracies in drug responses, such as tachycardia effects in hiPSC-CMs by calcium channel blockers (Zeng et al., 2019). Moreover, electrical propagation plays a vital role in cell–cell electrical coupling *via* gap junctions at the tissue level. The structural components of the intercalated disc complex, such as desmosomes, N-cadherin-mediated adherens junctions, Nav1.5, and connexin 43 (Cx43; encoded by GJA1), are circumferentially polarized in mature CMs, but not in immature CMs. This polarization does not occur in hPSC-CMs, but 3D tissue architecture can induce polarization by cell elongation and cyclic mechanical stretching (Salameh et al., 2010; Zhang et al., 2017b). Thus, the ability to mimic the *in vivo* environment to recapitulate mature CMs is important for obtaining more accurate drug responses and improving drug discovery applications.

## The Necessity of Human Engineered Heart Tissues as 3D Cardiac Tissues Models

**FIGURE 2:**
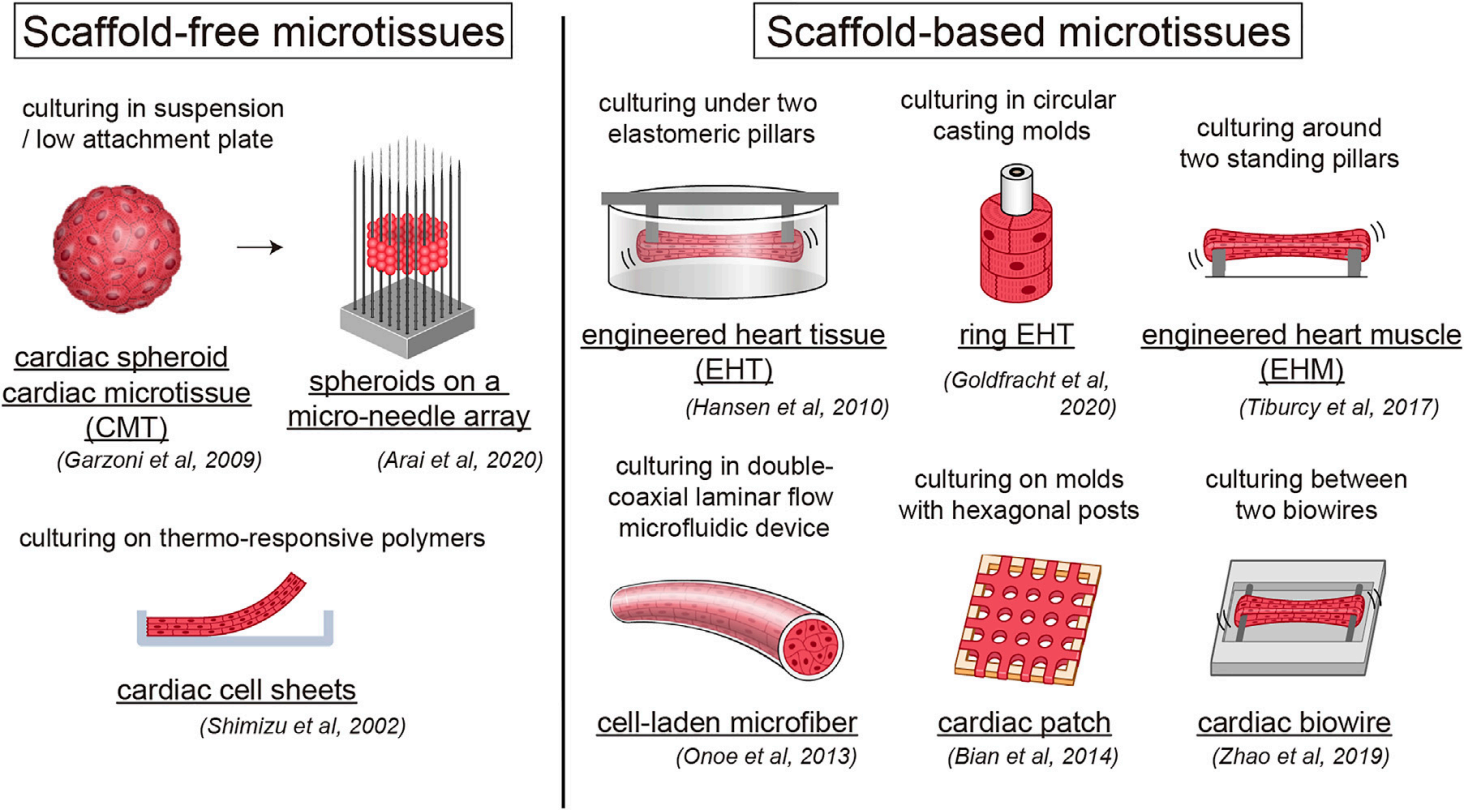
Human engineered heart tissue models with or without scaffold. Overview of scaffold-free and scaffold-based human engineered heart tissue models.

While larger 3D differentiation systems are increasingly utilized to generate sufficient CMs for transplantation studies, the conventional two-dimensional (2D) cell culture system is suitable for high-efficiency generation of pure hiPSC-CMs by maintaining a pluripotent state and high efficiency of differentiation (Tohyama et al., 2017). We also developed a 2D culture system using multilayer (10-layer) culture plates with active gas ventilation, which enabled the generation of large quantities of CMs (>1.0 × 10^9^ CMs). However, standard 2D cell culture cannot simulate the *in vivo* dynamic physical and environmental cues required to induce physiological hypertrophy, failing to recapitulate some heart disease phenotypes. *In vitro* approaches to cardiac maturation known to be effective include long-term culture, extracellular matrix (ECM) viscoelasticity, mechanical strain, electrical stimulation, triiodothyronine (T3), glucocorticoids, insulin-like growth factor 1 (IGF1), alterations in cellular energy sources (fatty acids), oxygen, peroxisome proliferator-activated receptors (PPARs), lethal-7 family miRNAs, estrogen-related receptor (ERR), and co-culture with non-CMs (Chan et al., 2013; Chun et al., 2015; Herron et al., 2016; Hu et al., 2018; Kroll et al., 2017; Lundy et al., 2013; Miki et al., 2021; Murphy et al., 2021; Parikh et al., 2017; Rog-Zielinska et al., 2015; Yang et al., 2014; Yang et al., 2019; Yoshida et al., 2018) (Figure 1). Notably, 3D culture systems can provide cellular elements, ECM scaffolds, and fluidic microenvironments, recapitulating cell-cell interactions *in vivo* and ideal maturation (Lundy et al., 2013; Mccain et al., 2014) (Table 1). Moreover, mechanical load on CMs enhances their maturation by auxotonic contraction, stretching, and afterload in the tissue. Accordingly, hydrogel scaffold-based 3D cardiac models such as engineered cardiac/heart tissues (ECT/EHT), engineered human myocardium (EHM), biowires, and HoC have advantages in that they are ideal reproducible 3D cardiac models with a unidirectional force of contraction, whereas scaffold-free 3D models such as cardiac spheroids, cardioids, cardiac microtissues (CMTs) and cell sheets are self-assembled, easy to fabricate, and do not require specific equipment (Hansen et al., 2010; Nunes et al., 2013; Onoe et al., 2013; Richards et al., 2017; Shimizu et al., 2002; Tiburcy et al., 2017; Zhang et al., 2017a; Zimmermann, et al., 2002). These methods have been developed by various laboratories to acquire maturity and are named in various ways (Figure 2). Moreover, scaffold-based 3D cardiac models with longitudinal stretching can be used to calculate the force of tissues quantitatively by measuring the distance between the points to which the scaffold was attached. In this review, we introduce how each 3D model has been developed to utilize its characteristics.

**TABLE 1:** Features of human heart models *in vitro*.

Construct		Composition and Description	Advantages	Disadvantages	Maturation Status					References
					Type of Analysis (AP Analysis)	Sarcomere	Contractility/CM Size	Ca Handling	Electrophysiology	
2D CMs		・CMs grown in plates or wells in culture	・ease of preparation	・immature CM phenotype	single-cells/sharp-electrode p.c. monolayer cells/whole-cell p.c.	sarcomere length; 1.65/1.81 μm (early/late phase) myofibrils are poorly organized, scattered across the cytoplasm	CM size; 480/1716 μm^2^ (early/late phase)	INa density; −10.3 pA/pF	RMP; −57/−68 mVMax dV/dt; 44/189 V/s	
		・thin layers or sheet constructs	・amenable to high-throughputs	・insufficient influence of non-CMs and the 3D environment					APD90; 146/189 msAPD50; 87/88 ms (early/late phase)	Lundy et al. (2013) Lemoine et al. (2017)
		・micropatterned to form rectangles	・measures of impulse propagation and arrhythmias・matured by media, patterning extracellular matrix manipulation	・unable to recapitulate some heart disease phenotypes						
2D CMs with non-CMs		hPSC-CMs + fibroblasts, ECs, and other cells	・mimicking the cellular composition of the heart	・insufficient influence of the 3D environment	single-cells/-	filament length ↑	CM size; 1,483/2,720 μm^2^ (CM/CM + MSC)	nd	nd	Yoshida et al. (2018)
			・recapitulating cell-cell interactions・enhanced CM maturation	・optimal components and composition ratio are still unknown						
3D CMs	cardiac spheroid cardiac microtissue (CMT) cardiac organoid/cardioid	hPSCs ± fibroblasts/ECs with self-assembly	・amenable to high-throughputs (low-cost, simplicity, and small numbers)	・unable to measure force	whole tissues/-	sarcomeres in Z-line width ↑ (organoids > spheroids)	nd	nd	nd	Richards et al. (2017)
			・mimicking the 3D cardiac environment	・difficult to assess EP with MEA	isolated cells/sharp-electrode p.c.	sarcomere length; 1.7–1.9 μm	nd	nd	RMP; −70 mV Max dV/dt; 150 V/s APD90; 250 ms	Giacomelli et al. (2020)
				・non-linear cell alignment						
	engineered cardiac/heart tissues (ECT/EHT)	・hPSC-CMs ± fibroblasts/ECs + scaffolds	・auxotonic contraction, stretching, and afterload	・real adult CM phenotypes have not been recapitulated	isolated cells/whole-cell p.c.	nd	nd	INa density; −18.5 pA/pF	RMP; −74 mV Max dV/dt; 219 V/s	Lemoine et al. (2017)
			・good electrical coupling (adaptable to pacing control)	・requiring large cell numbers and preparation with devices						
	engineered human myocardium (EHM)	・cast in a mold with 2 elastomeric pillars	・able to easily generate with low variation	・risk of breaking	isolated cells/whole-cell p.c.	clear Z-lines, I-bands, and A-bands, no M-line, sarcomere length; 2.3 μm	contractility; 0.3 mN	Ca transient; Enhanced	RMP; −60 mV Max dV/dt; 148 V/s APD90; 110 ms APD50; 60 ms	Mills et al. (2017)
			・measures of force, AP	・unequal distribution of cells						
			・natural alignment of cells/sarcomeres	・low to moderate throughput	isolated cells/whole-cell p.c.	orderly register of A-bands, I-bands, Z-lines, and M-lines. sarcomere length; 2.2 μm	contractility; 3 mN/mm^2^ (6Hz). CM size; 1,500 μm^2^	Ca transient; Enhanced	RMP; −70 mV Max dV/dt; 23 V/s APD90; 500 ms	Ronaldson-Bouchard et al. (2018)
			・further enhanced CM maturation					Ca storage/SR release; Enhanced		
	cardiac biowire	・hPSC-CMs ± fibroblasts/ECs + scaffolds	・high longitudinal tension	・requiring preparation with instruments	whole tissues/sharp-electrode p.c.	nd	contractility; 16 μN (3 Hz)	IKr; 0.8 pA/pF IK1 density; 1.5 pA/pF	RMP; −60–70 mV	Zhao et al. (2019)
		・cast in PDMS channels with an anchored surgical suture in line with the channel	・able to quantify tension	・absorb fluorescent hydrophobic compounds					Max dV/dt; 20 V/s APD90; 100 ms	

AP, action potential; CM, cardiomyocytes; EC, endothelial cells; EP, electophysiology; hPSC-CM, human pluripotent stem cell-derived cardiomyocyte; MEA, multielectrode array; MSC, mesenchymal stem cells; p.c., patch clamp; RMP, resting membrane potential; 3D, three-dimensional.

Engineered heart tissues consist of hydrogels and cardiac cells cast in a mold with two elastomeric pillars, which were first developed by Zimmermann et al. (2002). These researchers developed highly differentiated 3D cardiac tissues with neonatal rat CMs and fibrin gels and represent a promising method for *in vitro* cardiac function research and tissue replacement therapy (Hansen, et al., 2010; Zimmermann, et al., 2002). Subsequently, they demonstrated that the maturation of hiPSC-derived EHT can be achieved by electrical and mechanical stimulation. Hirt et al. found that hiPSC-derived EHT can be further matured with a denser cellular network, a well-organized ultrastructure including M-bands and connexins, and increased electrophysiological activities by continuous electrical stimulation (Hirt et al., 2014). Godier-Furnémont et al. also verified that concurrent electromechanical stimulation at physiological frequency induces a positive force–frequency relationship with functional maturation in the EHM (Godier-Furnémont et al., 2015). Leonard et al. (2018) demonstrated that functional maturation of hiPSC-CMs in EHTs requires a suitable afterload. Saleem et al. confirmed the positive relationship between calcium frequency and force frequency using a calcium-sensitive fluorophore and EHTs (Saleem et al., 2020). Recently, force-velocity curves and work loops with human EHTs have been applied to analyze length- and load-dependent muscle performance and diastolic function (Sewanan et al., 2021). These scaffold-based 3D hiPSC-CM tissues are useful not only for disease modeling but also for drug screening or cardiotoxicity assays. Lemoine et al. (2018) demonstrated that hiPSC-derived EHT, such as Purkinje fibers, showed a lower repolarization reserve by AP prolongation and early afterdepolarizations (EADs) compared to human LV working myocardium and that they can be applied as a sensitive and specific human-based model for AP and arrhythmia studies. Patient-specific EHTs with hereditary diseases, such as ion channelopathy and structural cardiomyopathy, and EHTs with chronic nonhereditary cardiomyopathy induction have been developed to date, which will be discussed further below. This research has the potential to be combined with gene editing, which may lead to improved personalized or precision medicine.

Their 3D structure facilitates the self-organization of CMs into more mature structures. Li et al. produced a device comprising a low-attachment substrate on which hiPSC-CMs can spontaneously organize into a 3D tissue ring (Li Junjun et al., 2020; Zhang et al., 2020). The 3D tissue ring can spontaneously generate reentrant waves without external stimulation, and rapid pacing enhances autonomous maturation of CMs, indicating their potential use as a model in arrhythmia studies.

Furthermore, it was reported that rapid electrical stimulation promotes the maturation of 3D tissues near the adult level. Ronaldson-Bouchard et al. reengineered the hanging pillar model to improve the alignment and centralization of force through the center of the tissue, which enabled the development of a more mature CM tissue-like EHT (Ronaldson-Bouchard, et al., 2018). An electrical pacing stimulation that gradually increased to 6 Hz enabled much greater maturation, with sarcomere lengths of 2.2 μm and remarkably mature ultrastructural morphology, calcium handling, and gene expression, although maturation in metabolism and electrophysiology has not been accomplished. In addition, they and Zhao et al. created a platform using a biowire system with atrial and ventricular hiPSC-CMs that showed distinct chamber-specific drug responses and gene expression (Zhao et al., 2019). However, unlike PDMS, this chip did not absorb fluorescent hydrophobic compounds, thereby complicating the interpretation of both long-term and short-term drug screening studies using conventional cardiac wire methods. These results pave the way for more reliable high-throughput screening methods using 3D cardiac tissues.

A decrease in necessary ingredients, and consequently costs, manipulation, and contamination risk, has been achieved by using more mini-scale and standing pillars. Mills et al. miniaturized the model to fit into 96-well plates, which can be utilized for high-throughput drug screening (Mills et al., 2017; Mills et al., 2019). They used this engineered cardiac muscle model to screen for cardiac maturation conditions and found that simulating the postnatal switch in metabolic substrates from carbohydrates to fatty acids induced a switch in metabolism, DNA damage response, and cell cycle arrest in hPSC-CMs (Mills, et al., 2017). Other groups have also validated the contractile force response of cardiac micro-rings in a 96-well-based array using selected cardiotropic compounds with known effects (Afshar et al., 2020; Thavandiran et al., 2020). Dostanic et al. miniaturized the standing pillar design and confirmed accurate measurements of contractile forces at the mini-scale (Dostanic et al., 2020).

Another strategy to generate 3D tissues *in vitro* is to use decellularized whole hearts while leaving the vasculature intact. Human decellularized cardiac tissue has been shown to support the viability and differentiation of mouse iPSCs and ESCs into CMs (Oberwallner et al., 2015). Lee et al. engineered components of the human heart at various scales, from capillaries to the full organ, by 3D-bioprinting with freeform reversible embedding of suspended hydrogels, which represents a potential for decellularized hydrogels (A Lee et al., 2019). Decellularized porcine myocardium has also been used as a scaffold for EHTs, yielding robust EHTs that are suitable for several biomechanical assays (Schwan et al., 2016; Ng et al., 2020).

Arai et al. developed a bio-3D printer technology and fabricated scaffold-free cellular constructs with 3D-laminating spheroids on a micro-needle array (Arai et al., 2020; Kawai et al., 2021). They generated an array with a 3D design by arranging the micro-needles to line up with the spheroids, revealing a robust assessment of contractile properties and drug response.

3D cultures of multiple cell types, so-called organoids, can imitate the *in vivo* tissue, structure, and function of an organ. Richards et al. (2017) developed scaffold-free hiPSC-derived cardiac organoids (COs) that structurally and functionally resemble the lumenized vascular network in the developing myocardium, enabling the optimization of the cellular, matrix/material, and additional factors necessary for heart development. They also modeled myocardial infarction (MI) using COs that recapitulated their pathological characteristics, such as fibrosis, metabolic shift, and calcium-handling properties (Richards et al., 2020). Buono et al. (2020) produced COs derived from HCM patients to confirm the significant phenotypes of human HCM hearts. Notably, rather than mixing components, Lee et al. developed a method to generate self-organizing COs in the presence of the laminin-entactin complex and fibroblast growth factor 4 from mouse embryonic stem cell-derived embryoid bodies (Lee et al., 2020). They not only presented a biomimetic model of the developing heart-like structure with a simple differentiation protocol, but also demonstrated a promising research tool with applications for human cells. Recently, Hofbauer et al. (2021) established self-organizing cardioids containing a cavity and recapitulated the heart lineage architecture. They demonstrated that cavity morphogenesis was governed by a mesodermal WNT-BMP signaling axis and required its target, HAND1, which is related to heart chamber defect development. They also recapitulated the fibrotic responses in the injury model by co-culture with epicardium aggregates. Thus, 3D models without scaffolds can also serve as a foundation for future translational research.

## The Optimization and Sophistication of 3D Cardiac Tissues Models

Scaffold hydrogels and ECM are important for the development of 3D tissues. Several types of collagen, fibronectin, and elastin are most common in the human heart, and these ECM components closely interact with cells by providing growth factors such as integrins to their receptors and sensing and transducing mechanical signals, leading to cell growth, morphology, function, migration, survival, gene expression, and differentiation (Rozario and Desimone, 2010; Johnson et al., 2016; Bildyug 2019). To date, various hydrogel mixtures have been used to fabricate EHT, and the most commonly used hydrogels with EHT are the natural ECM protein collagen type I, Matrigel, which is known as ECM from Engelbrecht-Holm-Swarm tumors in mice containing principally collagen IV, laminin, and fibrin, which is useful for coagulation (Hansen, et al., 2010; Zimmermann, et al., 2002).

The quantity and type of non-CMs in an *in vitro* co-culture system are also critical to yield functional hiPSC-CMs. ECM components mainly consist of non-CMs and are required for optimal heart tissue organization. Non-CMs, such as endothelial cells (ECs), cardiac fibroblasts (CFs), and leukocytes, account for 70% of the total cardiac cell population (Bergmann et al., 2015; Pinto et al., 2016). While considerable effort has been made to generate efficient differentiation protocols and enrich for hPSC-CMs, the addition of fibroblasts has improved cell viability, self-organization, and contractility, and inclusion of ECs to these CMTs resulted in the formation of vascular-like structures (Garzoni et al., 2009; Noguchi et al., 2016). Non-CMs are important for ECM remodeling and the fabrication process from hiPSC-CMs to EHT. Interestingly, Tiburcy et al. (2017) confirmed the influence of fibroblasts on tissue creation. Fibroblasts are vital for hydrogel condensation, with a ratio of 70% hiPSC-CM and 30% fibroblasts being optimal for the force of contraction. Although some studies have shown that hPSC-CMs without additional fibroblasts can produce functional 3D models, few residual non-CMs or hydrogels are likely to contribute to tissue formation and organization (Mills et al., 2017; Goldfracht et al., 2020). Multiple studies have shown that even without the intentional addition of non-CMs, CD31^+^ ECs and CD90^+^ stromal cells can be detected in the tissue (Abilez, et al., 2018). This may be due to the intermediates and residual cardiac progenitors that arise during the differentiation process. Giacomelli et al. developed 3D CMTs composed of 70% CMs, 15% ECs, and 15% CFs, revealing that CFs and ECs induce hiPSC-CM maturation and that Cx43 gap junctions form between CFs and CMs (Giacomelli, et al., 2020). Moreover, to mimic more complex heart diseases, it may be important to add inflammatory components (e.g., macrophages and inflammatory proteins) or vascularization. Bailey et al. developed EHTs containing hPSC-CMs, 5% CFs, and 10% monocyte-derived macrophages, and showed that infection with SARS-CoV-2 induced typical features of myocarditis, including CM death, cardiac dysfunction, and impaired immune cell activation (Bailey et al., 2021). Macrophages in engineered constructs have been shown to serve not only in immunological responses but also in tissue maturation, repair, regeneration, and vascularization (Suku et al., 2021). Mills et al. demonstrated that proinflammatory factors drive systolic and diastolic cardiac dysfunction in hPSC-CM-derived COs containing 20% ECs, recapitulating SARS-CoV-2 infection (Mills et al., 2021). Silva et al. demonstrated that the presence of endoderm tissue (gut/intestine) in COs contributes to the development of cardiac tissue features, including CM expansion, compartmentalization, enrichment of atrial/nodal cells, myocardial compaction, and fetal-like functional maturation (Silva et al., 2021). These results indicate the importance of multi-tissue interactions during development and maturation. Further assessment of more correct tissue components and substrate stiffness is required for physiologically relevant cardiac responses (Mills, et al., 2017; Ribeiro et al., 2015).

Most EHTs have been cultured in glucose-based media, but culture medium with low carbohydrates, low insulin, and palmitate without serum has been shown to induce CM maturation, mimicking the metabolic switch in heart development. Mills et al. demonstrated that under these conditions, key proliferation pathways such as β-catenin and yes-associated protein 1 (YAP1) were repressed, force increased, Ca^2+^ handling was more mature, mitochondrial mass was increased, and overall organization improved (Mills, et al., 2017). Passive stretch in 3D culture also causes a switch from glycolysis to oxidation in hiPSC-derived EHTs (Ulmer et al., 2018). Feyen et al. developed a low-glucose, high-oxidative substrate medium (maturation medium; MM) that increased the functional and structural maturation of hiPSC-derived EHTs and improved the stability of EHTs (Feyen et al., 2020).

## Patient-specific iPSC-CMs as Disease Models

Disease models can be recapitulated *in vitro* using patient-specific hiPSC-CMs. These models can be classified as hereditary and represent a type of cardiomyopathy, as reviewed below (Table 2).

**TABLE 2:** Lists of disease-specific hPSC-CM models

Disease Model Categories			Disease Phenotype/Features	Causative Genes	References	3D Models
Inherited Cardiomyopathy						
Ion Channelopathy	LQTS	LQTS1	QT prolongation can cause lethal arrhthmia	*KCNQ1*	Moretti et al. (2010)	−
					Egashira et al. (2012)	−
					Takaki et al. (2019)	−
		LQTS2		*KCNH2*	Chang et al. (2021)	−
		LQTS3		*SCN5A*	Malan et al. (2016)	−
					Okata et al. (2016)	−
					McKeithan et al. (2020)	−
		LQTS7		*KCNJ2*	Kuroda et al. (2017)	−
		LQTS8		*CACNA1C*	Yazawa et al. (2011)	−
		LQTS14		*CALM1*	Rocchetti et al. (2017)	−
		LQTS15		*CALM2*	Limpitikul et al. (2017)	−
		LQTS16		*CALM3*	Wren et al. (2019)	−
	SQTS	SQTS1	shortened QT interval	*KCNH2*	El-Battrawy et al. (2018)	−
	BrS		depolarization, repolarization disorders	*SCN5A*	Li Junjun et al. (2020)	−
	CPVT	CPVT1	abnormal Ca2+ leakage	*RYR2*	Park et al. (2019)	EHT
Structural Cardiomyopathy	HCM		myocardial-related protein disorders cause cardiac dysfunction and cardiac hypertrophy	*MYH7*	Lan et al. (2013)	−
				*MYBPC3*	Tanaka et al. (2014)	−
				*MYH7*	Mosqueira et al. (2018)	EHT
				*BRAF*	Cashman et al. (2016)	ECT
				*MYBPC3*	Wijnker et al. (2016)	EHT
				*PRKAG2*	Hinson et al. (2016)	CMT
				*ACTN2*	Prondzynski et al. (2019)	EHT
				*MYBPC3*	Flenner et al. (2021)	EHT
				*MYBPC3*	Tsan et al. (2021)	Micron-scale bundles
				*TNNT2*	Cai et al. (2020)	−
	DCM		myocardial-related protein disorders cause cardiac dysfunction, enlarged chamber size and thinner chamber walls	*TNNT2*	Sun et al. (2012)	−
				*TNNT*	Dai et al. (2020)	EHM
				*TITIN*	Hinson et al. (2015)	CMT
				*TNNT2*	Pettinato et al. (2020)	CMT
				*PLN*	Stillitano et al. (2016)	ECT
				*PLN*	Feyen et al. (2021)	EHT
				*RBM20*	Streckfuss-Bomeke et al. (2017)	EHM
	ACM		disorders of cytoskeleton and adhesion related protein cause cardiac dysfunction and arrhthmia	*PKP2*	Ma et al. (2013)	−
				*PKP2*	Kim et al. (2013)	−
				*PKP2*	Kohela et al. (2021)	−
				*DSP*	Bliley et al. (2021)	EHT
	DMD		disorders of cytoskeleton cause progressive cardiac muscle dysfunction	*DMD*	Guan et al. (2014)	−
				*DMD*	Kyrychenko et al. (2017)	−
				*DMD*	Long et al. (2018)	EHM
Nonhereditary Cardiomyopathy						
	HFrEF		a genetic term of heart failure with reduced ejection fraction (<40%)		Tiburcy, et al. (2017)	EHM
					Fiedler et al. (2019)	−
					Iseoka et al. (2021)	−
	Myocardial ischemia		reduced coronary flow cause CM death and fibrosis		Richards, et al. (2020)	COs
					Sebastião et al. (2020)	Aggregates
					Knight et al. (2021)	Micropatterned
	Tachcardia induced cardiomyopathy		tachycardia cause cardiac dysfunction		Lemme et al. (2020)	EHT
	Diabetic cardiomyopathy		DM-related structural, functional disorders		Drawnel et al. (2014)	−

ACM, arrhythmogenic cardiomyopathy; BrS, brugada syndrome; CM, cardiomyocytes; COs, cardiac organoids; CPVT, catecholaminergic polymorphic ventricular tachycardia; DCM, dilated cardiomyopathy; DM, diabetes mellitus; DMD, duchenne muscular dystrophy; HCM, hypertrophic cardiomyopathy; LQTS, long QT syndrome; SQTS, short QT syndrome.

### Inherited Cardiomyopathy

#### Ion Channelopathy

The hiPSC-CM disease models of ion channelopathies are attractive due to the advantage of being able to measure single-cell electrophysiological profiles, like APD. Cardiac channelopathies related to arrhythmias are predicted to be abnormalities of APD, synchronization, or propagation. Long QT syndrome (LQTS) is the most common ion channelopathy, with a prevalence of 1 in 2000 (Schwartz et al., 2009). Long QT syndrome is characterized by prolonged repolarization and susceptibility to syncope and sudden cardiac death (SCD) due to polymorphic ventricular tachycardias such as torsade de pointes (TdP) (Modell and Lehmann, 2006). More than 15 different ion channel mutations related to the cardiac voltage-gated potassium (Kv) channel and the voltage-gated sodium (Nav) channel have been reported as causative genes, although mutations in *KCNQ1*, *KCNH2*, and *SCN5A* account for the majority of them (Brewer et al., 2020). LQTS type 1 (LQTS1) is caused by mutations in *KCNQ1*; Moretti et al. (2010) first introduced LQTS1 hiPSC-CM disease modeling. We also showed that patient-derived iPSCs with a mutation in *KCNQ1* could recapitulate the disease phenotype in a case of sporadic LQTS1 (Egashira et al., 2012). Takaki et al. developed different disease-specific hiPSC lines with *KCNQ1* mutations and demonstrated that this model precisely reflects the disease phenotype, with slow outward I_Ks_, abnormal channel activities, and increased arrhythmogenicity (Takaki et al., 2019). LQTS2 occurs due to mutations in *KCNH2*, known as a human ether-à-go-go-related gene (*hERG*), which conducts I_Kr_ (Van Den Boogaard et al., 2019). Itzhaki et al. revealed that LQTS2 disease models show a significant prolongation of the APD due to a reduction in I_Kr_ and evaluated the effects of some pharmacological agents on the disease phenotype (Itzhaki et al., 2011). Chang et al. (2021) used CRISPR/Cas9 editing to generate a KCNH2-KO hiPSC-CM model and showed that the model exhibited QT prolongation, irregular rhythm, and sensitivity to ion channel blockers such as L-type Ca channel blockers. Bellin et al. (2013) demonstrated that correction of the *KCNH2* mutation normalized the I_Kr_ current conducted by the hERG channel and APD. LQTS3 occurs due to mutations in *SCN5A*, which is known to mediate fast Nav1.5 sodium channel inactivation. Malan et al. (2016) generated hiPSC-CMs with an *SCN5A* mutation in a patient with LQTS3. They recapitulated the pathognomonic electrophysiological features such as accelerated recovery from inactivation of Nav1.5, AP prolongation, and EADs, particularly at low stimulation rates, leading to arrhythmogenicity. McKeithan et al. (2020) used large-scale functional screening of LQTS3 hiPSC-CMs to direct the chemical optimization of mexiletine, an antiarrhythmic drug for this disease, and identified four new optimized analogs that simultaneously improved potency and decreased undesired proarrhythmic liability. Less common LQTS has also been studied in patient-specific hiPSC-CMs such as LQTS7 (Andersen-Tawil syndrome; mutations in *KCNJ2*), LQTS8 (Timothy syndrome; mutations in *CACNA1C*), LQT14 (mutations in *CALM1*), LQT15 (mutations in *CALM2*), and LQT16 (mutations in *CALM3*) (Yazawa et al., 2011; Kuroda et al., 2017; Limpitikul et al., 2017; Rocchetti et al., 2017; Wren et al., 2019).

Short QT syndrome (SQTS) is a rare inherited channelopathy with a prevalence estimated to be less than 1 in 10,000, which causes both atrial and ventricular tachyarrhythmias, syncope, and SCD (Fan et al., 2021). Six genes encoding potassium channels (*KCNH2*, *KCNQ1*, *KCNJ2*) and calcium channels (*CACNA1C*, *CACNB2*, *CACNA2D1*), which are causative genes of other ion channelopathies, have been found to be associated with SQTS (Mazzanti et al., 2014). An hiPSC model of SQTS1 using hiPSC-CMs derived from a patient with a KCNH2 mutation recapitulates the single-cell phenotype of SQTS (El-Battrawy et al., 2018). However, the diagnostic and treatment approaches are still challenging due to their low prevalence, and even the mechanisms of arrhythmogenesis in SQTS are not well understood (Fan, et al., 2021).

Brugada syndrome (BrS) is an autosomal dominant inherited channelopathy associated with a unique ECG pattern of coved-type ST-elevation followed by T-wave inversion in the right precordial leads, occurring spontaneously or under sodium channel blocker challenge (Brugada and Brugada, 1992). BrS can cause ventricular fibrillation and SCD in young adults with structurally normal hearts, with a prevalence estimated to be 1 in 2000–5,000 with a male dominance (Brugada et al., 2018). Loss of function mutations in *SCN5A* accounts for 30% of BrS cases, although 19 genes related to Na, K, or Ca channels have been reported to be associated with BrS (Tse et al., 2016). The abnormality of SCN5A in the presence of a transmural voltage gradient by heterogeneous transmural distribution of I_to_ accentuates the AP notch and loss of spike and dome morphology, leading to phase 2 reentry and polymorphic ventricular tachycardia (Yan and Antzelevitch, 1996). Okata et al. produced hiPSC-CMs from a patient with a mixed phenotype of LQTS3 and BrS with a mutation in *SCN5A* and demonstrated that hiPSC-CMs recapitulate the electrophysical phenotype of LQTS3 but not that of BrS (Okata et al., 2016). They hypothesized that intrinsic cell factors underlie the differential manifestation of young-onset LQTS3 and adult-onset BrS phenotypes. Li et al. recently successfully exhibited reduced AP upstroke velocity, conduction slowing, and depolarization and repolarization disorders by using hiPSC-CMs from BrS patients with an *SCN5A* mutation (Li Wener et al., 2020).

Catecholaminergic polymorphic ventricular tachycardia (CPVT) is another common inherited channelopathy characterized by emotion- and exercise-induced polymorphic ventricular arrhythmias, leading to SCD. Generally, CPVT is divided into CPVT1, caused by mutations in *RYR2* encoding the cardiac ryanodine receptor, and the less common CPVT2, caused by a mutation in *CASQ2* encoding cardiac calsequestrin (Lieve et al., 2016). These mutations cause abnormal Ca^2+^ leakage from the sarcoplasmic reticulum (SR), leading to cytosolic Ca^2+^ overload, which subsequently delays afterdepolarizations and triggers ventricular arrhythmias. Park et al. (2019) developed hiPS-CMs with a mutation in *RYR2* using CRISPR/Cas9 editing and effectively recapitulated the CPVT1 features, including the induction of arrhythmias. They demonstrated that activation of Ca^2+^/calmodulin-dependent protein kinase was a key factor for provoking arrhythmias, highlighting a molecular pathway connecting β-adrenergic stimulation to arrhythmogenesis.

#### Structural Cardiomyopathy

Hypertrophic cardiomyopathy (HCM) is the most common inherited cardiomyopathy with morphological abnormalities, with an estimated prevalence of 1 in 200–500 (Maron 2002). Disorganized cellular architecture, myocardial scarring, and expanded interstitial collagen serve as ventricular arrhythmogenic substrates, and the conversion to ventricular fibrillation causes SCD in many patients (Geske et al., 2018). Over 1,500 mutations related to HCM have been identified, most of which are associated with sarcomere genes, resulting in the dysregulation of CM contraction and relaxation. One study showed that around 70% of HCM patients had *MYH7* (encoding β-myosin heavy chain) or *MYBPC3* (myosin-binding protein) mutations, which negatively affect force generation (Marian and Braunwald, 2017). Less common mutations are associated with other sarcomere genes, such as actin (*ACTC*), cardiac troponin T (*TNNT2*), myosin light chain (*MYL2*), and cardiac troponin I (*TNNI3*), or non-sarcomere genes such as ion channels, Z-disc genes, and membrane transporters. Lan et al. generated patient-specific hiPSCs from a ten-member family cohort with a hereditary HCM missense mutation (A663H) in *MYH7*, which recapitulated key features of the HCM phenotype, such as disorganized sarcomeres and aberrant Ca^2+^ handling (Lan et al., 2013). Tanaka et al. generated patient-specific hiPSCs from three patients with HCM to characterize pathological phenotypes. The phenotype of myofibrillar disarray and contractile vector variability in HCM were mild under baseline conditions, but endothelin-1 strongly induced the pathological phenotypes (Tanaka et al., 2014). Mosqueira et al. used CRISPR/Cas9 editing to develop HCM disease modeling with site-directed homozygous or heterozygous variants (Mosqueira et al., 2018). Such cell-based models provide a novel *in vitro* model for specifying pathogenesis and developing therapeutics for cardiomyopathies. There are advantages to using 3D models to elucidate mechanisms compared to 2D models, as they can better reflect and mimic cell-cell interactions at the tissue level. Cashman et al. (2016) generated ECTs from patients with cardio-facio-cutaneous syndrome with *BRAF* mutations (encoding a serine/threonine kinase); this 3D model recapitulated the phenotypes of HCM, offering a new *in vitro* model to study intrinsic mechanisms and screen new therapeutic approaches. Wijnker et al. compared the pathomechanisms of a truncating mutation (c.2373_2374insG) and a missense mutation (c.1591G > C) in *MYBPC3* by EHT contractile function (Wijnker et al., 2016). Hinson et al. revealed that hiPSC-CMs with a *PRKAG2* mutation recapitulated hypertrophy and glycogen accumulation due to AMPK activation and used CMT modeling to show that AMPK activation attenuates fibrosis and adverse remodeling in HCM (Hinson et al., 2016). Prondzynski et al. demonstrated that hiPSC-CM-derived EHTs with an *ACTN2* mutation (p.T247M) recapitulated the phenotype of HCM, such as hypertrophy, altered calcium response, hypercontractility, and sarcomeric disarray, and showed the efficacy of L-type calcium channel inhibition (Prondzynski et al., 2019). After demonstrating the efficacy with diltiazem in EHTs, the drug improved the electrical phenotype of HCM-affected family members. Flenner et al. generated hiPSCs with an HCM mutation (homozygous *MYBPC3* mutant; MYBPC3hom) and the hiPSC-CM-derived EHTs harboring this mutation exhibited lower K^+^ current protein levels, force, beating frequency, and relaxation time, highlighting the application of not only force measurements but also arrhythmia susceptibility (Flenner et al., 2021). Tsan et al. generated micron-scale cardiac muscle bundles from hiPSC-CMs with a deletion of the *MYBPC3* promoter, and demonstrated that contractile velocities were higher while relaxation velocities were lower, recapitulating the kinetics of HCM (Tsan et al., 2021). Cai et al. generated *TNNT2* R92Q mutant hESC-derived CMs, implying increased calcium sensitivity and contractility, and demonstrated that they exhibited efficient responses to heart-related pharmaceutical agents (Cai et al., 2020).

Dilated cardiomyopathy (DCM) is the second most common cause of HF following coronary artery disease (CAD), which accounts for 30–40% of HF cases, and is the primary indication for heart transplantation, with a varying prevalence of 1 in 250–2,700 worldwide (Haas et al., 2015; Mcnally and Mestroni, 2017). DCM is primarily caused by sarcomere gene mutations. DCM-diseased hearts are characterized by an enlarged chamber size and thinner chamber walls, with volume overload and impaired systolic function, subsequently leading to progressive HF. DCM-diseased hearts show structural abnormalities such as fibrosis and scarring, which give rise to defects in conduction and refractoriness, leading to a substrate for reentrant arrhythmias (Wu et al., 1998). Over 50 different genes related to DCM have been identified, and approximately 20–25% of patients with DCM have the most prevalent mutations in *TTN* (Herman et al., 2012; Nomura 2019). Sun et al. first generated DCM hiPSC-CMs with a mutation in *TNNT2*, recapitulating the morphological and functional phenotypes of DCM (Sun et al., 2012). They exhibit impaired Ca^2+^ management, including lower SR Ca^2+^ storage and decreased Ca^2+^ inflow, leading to reduced contractility and abnormal α-actin distribution, mimicking CMs in failing hearts of human and animal models. These abnormalities occurred when the mitochondria and SR were still immature in both DCM and control hiPSC-CMs, indicating that abnormalities in Ca^2+^ handling can occur at an early stage of heart development. Dai et al. demonstrated that the *TNNT* mutation destabilizes the molecular interactions of troponin with tropomyosin and limits the binding of PKA to sarcomeres (Dai et al., 2020). Hinson et al. used a CMT model to show that titin mutations cause DCM by breaking the linkages between sarcomerogenesis and adaptive remodeling (Hinson et al., 2015). This group and Pettinato et al. also found that HCM-associated *TNNT2* variants increased CMT contraction, while DCM-associated variants decreased contraction, both of which paralleled changes in myofilament calcium affinity (Pettinato et al., 2020). This revealed that transcriptomic changes directly correlate with sarcomere function and can be used to predict *TNNT2* variant pathogenicity, indicating that reclassification of *TNNT2* variants would improve cardiomyopathy risk determination and treatment responses in patients with these variants. Less common mutations are found in nuclear lamina, Nav channel α-subunit 5 (*SCN5A*), desmin (*DES*), phospholamban (*PLN*), Bcl2-associated athanogene 3 (*BAG3*), and RNA-binding motif protein 20 (*RBM20*) (Rosenbaum et al., 2020). EHTs derived from hiPSC-CMs with a *PLN* R14del mutation recapitulated muscle contractility (Stillitano et al., 2016). Feyen et al. developed hiPSC-CMs with a *PLN* R14del mutation using CRISPR/Cas9 genome editing, and recapitulated contractile dysfunction in EHTs (Feyen et al., 2021). They also observed elevations in endoplasmic reticulum (ER) stress and the unfolded protein response (UPR), and revealed a protective role of UPR activation by pharmacological modulation of the UPR pathway. EHMs derived from hiPSC-CMs with an RBM20 mutation revealed not only impaired active force generation but also a decrease in passive stress in the tissues (Streckfuss-Bomeke et al., 2017).

Arrhythmogenic cardiomyopathy (ACM), or arrhythmogenic right ventricular cardiomyopathy (ARVC), is another inherited disease that results in progressive loss of the myocardium. Patients are predisposed to a wide spectrum of clinical presentations, such as ventricular arrhythmia and SCD, which are associated with mutations in desmosome genes, such as plakoglobin (*JUP*), desmoplakin (*DSP*), plakophilin 2 (*PKP2*), desmoglein 2 (*DSG2*), and desmocollin 2 (*DSC2*) (Romero et al., 2013; Corrado et al., 2017). *PKP2* mutation is the most common pathogenic mutation found in ACM. HiPSC-CMs with a *PKP2* mutation were found to recapitulate key features of ACM, such as low β-catenin activity, abnormal nuclear translocation of junction plakoglobin, and reduced cell surface localization of desmosomes, presenting an adipogenic phenotype (Ma et al., 2013). Kim et al. reported that hiPSC-CMs with a *PKP2* mutation can recapitulate the phenotypes of ACM by co-activating PPAR-α/PPAR-γ pathways, both of which are responsible for metabolism (Kim et al., 2013). Kohela et al. differentiated hiPSC-CMs with a PKP2 mutation into epicardial cells, which showed spontaneous fibro-fatty cellular differentiation, and identified the transcription factor activating enhancer-binding protein 2α as a key trigger (Kohela et al., 2021). Bliley et al. (2021) differentiated hiPSC-CMs with a DSP mutation and developed a new dynamic EHT system that provides a mechanical preload and afterload to provoke the ACM disease phenotype of diastolic lengthening, reduction of desmosome counts, and reduced contractility. These results suggest that ACM requires stress conditions, complex 3D models, and non-CMs.

Duchenne muscular dystrophy (DMD) is a rare X-linked recessive disease with an incidence of 1 in 5,000 males. DMD patients are highly susceptible to mechanical stress and injury due to the deficiency of dystrophin protein (Mendell et al., 2012). Dystrophin is composed of the dystrophin-glycoprotein complex, which exists at the muscle sarcolemma and bridges the cytoskeleton and extracellular matrix, which maintains cellular stability on the inner side of skeletal and cardiac muscle cells (Fayssoil et al., 2010). Deficiency of dystrophin causes progressive muscle scarring, degeneration, and HF, leading to death. Guan et al. developed a model of DMD stem cell-derived CMs, which recapitulated abnormal Ca^2+^ handling, increased sensitivity to hypotonic stress, and altered contractile mechanics (Guan et al., 2014). Kyrychenko et al. used genome editing to develop DMD hiPSC-CMs, which exhibited excessive Ca^2+^ influx and increased sensitivity to hypotonic stress, generation of reactive oxygen species (ROS), and mitochondrial damage, leading to cell apoptosis, and demonstrated that deletion of exons in the DMD gene restored muscle function (Kyrychenko et al., 2017). Using a 3D EHM model, their group also demonstrated that modification of dystrophin with CRISPR/Cas9 is a promising approach for rescuing DMD by restoring CM contractility and calcium transients (Long et al., 2018).

### Chronic Nonhereditary Cardiomyopathy

Acquired cardiomyopathies are caused not by genetic modification but by drug and other environmental stimulations. Chronic HF is a progressive syndrome that results in a poor quality of life for patients, with an estimated prevalence of 1 in 120 individuals with HF. Chronic HF is caused by CVDs, including CAD and MI, and high blood pressure, leading to structural or functional changes in the heart (Mudd and Kass, 2008). Heart failure with reduced ejection fraction is defined as HF with a 40% or lower left ventricular ejection fraction and accounts for approximately 50% of all HF cases (Murphy et al., 2020). Heart failure with reduced ejection fraction hearts presents hypertrophy, a weaker force-frequency response, and decreased β-adrenergic sensitization. Tiburcy et al. generated EHM models of HF using chronic norepinephrine stimulation (Tiburcy, et al., 2017). This model showed not only pathological hypertrophy, cellular death, and impaired contractile function but also N-terminal pro B-type natriuretic peptide (NT-proBNP) release, which is used for the clinical diagnosis of HF. They provided a promising tool for HF modeling, drug screening, and tissue-based heart repair. Fiedler et al. (2019) developed a myocardial ischemia model exhibiting the most prevalent etiology of HF and evaluated optimized analogs by screening for cardioprotective activity. Iseoka et al. induced an HF model by TGF-β stimulation and detected anti-fibrotic drugs that decreased fibrotic ECM expression and improved contraction and relaxation using a cell-motion system (Iseoka et al., 2021).

Richards et al. demonstrated that COs experiencing hypoxia and chronic adrenergic stimulation with the neurotransmitter noradrenaline mimic the structure of the human heart after MI, recapitulating pathological metabolic shifts, fibrosis, and calcium handling in terms of transcriptome, structure, and function (Richards, et al., 2020). Sebastião et al. recapitulated hallmarks of acute MI, such as loss of CM viability, increased angiogenic potential, and secretion of key proangiogenic and proinflammatory cytokines, which recovered during the post-ischemia to post-reperfusion transition (Sebastião et al., 2020). Knight et al. revealed pathological hypertrophy with associated myofibril relaxation defects in response to a pro-hypertrophic agent by utilizing mature hiPSC-CMs with fatty acid-based medium and micropatterned plating (Knight et al., 2021). This report suggests that mature hiPSC-CMs may enable the pathogenesis of chronic late-onset HF.

Lemme et al. developed a unique chronic tachypacing hiPSC-CM-derived EHT model with an optogenetic approach and demonstrated its effective termination using ryanodine receptor stabilization, sodium, or hERG potassium channel inhibition (Lemme et al., 2020). This new model shows potential for the testing of antiarrhythmic drugs, which would increase our insight into treating ventricular tachycardia.

Drawnel et al. developed a phenotypic surrogate model of type 2 diabetic cardiomyopathy to recapitulate the phenotype of structural and functional disorders (Drawnel et al., 2014). Several of the identified compounds showed dysregulated calcium cycling and signaling as the cause of the phenotypic effects.

Thus, disease-specific iPSC-CMs, such as ion channelopathy, structural cardiomyopathy, metabolic cardiopathy, and chronic nonhereditary cardiomyopathy have been developed with gene editing in the context of reversing a pathological phenotype, and their maturation by 3D fabrication contributes to the reproducibility of the disease-specific iPSC-CMs. Moreover, these cells not only recapitulate diseases and help elucidate their underlying mechanisms, but also guide the medicinal chemical optimization of new or existing drugs. Indeed, they have facilitated drug discovery for the treatment of LQT3 and ischemia models (Fiedler, et al., 2019; McKeithan, et al., 2020). These results illustrate that hiPSC-CMs can facilitate the rapid medicinal chemical refinement of drugs to improve their therapeutic potential and reduce side effects. However, the disassembly and isolation of 3D constructs are required for precise evaluation of electrophysiology, and 3D heart models of ion channelopathies have not been fully developed, compared with those of structural cardiomyopathies (Table 2). Further applications of heart tissues with both maturation and confident evaluation systems are expected.

## The Roles of hiPSC-CMs in New Drug Development

**FIGURE 3:**
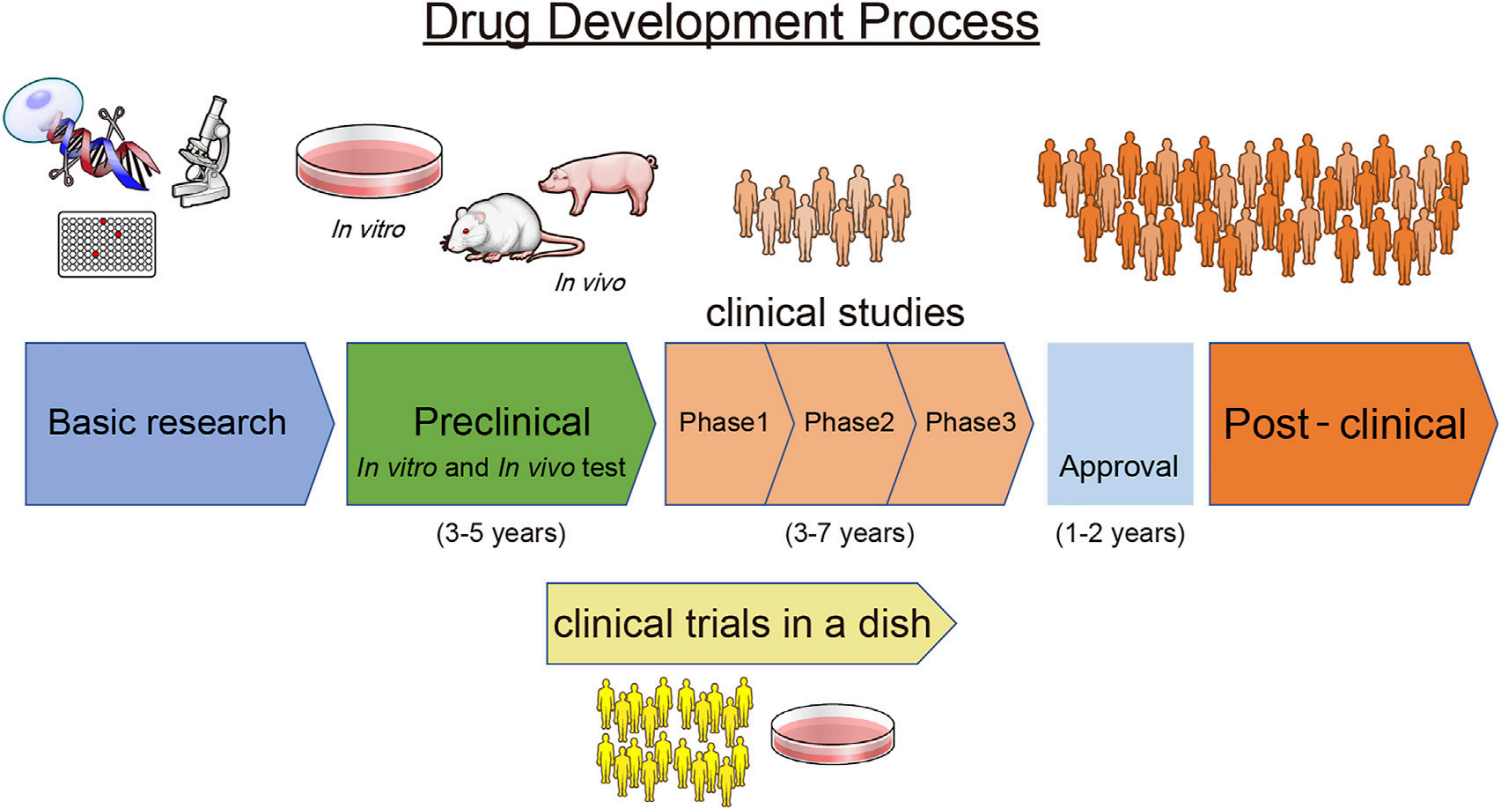
Drug Development Process. A scheme describing the drug development process.

During the preclinical stages of new drug development, it is important to fully assess the safety and pharmacokinetics of the drug. From the perspective of the time- and cost-intensive process of providing new candidate drugs to the market, removing failed candidates as early as possible is essential. Generally, the drug development process includes an early discovery phase, preclinical studies, clinical studies (phases I, II, and III), approval, and a post-marketing phase (Figure 3). As described above, disease-specific hPSC-CMs can aid in the elucidation of drug pathophysiology, selection of candidates, and assessment of efficacy. In addition, use of hPSC-CMs can be more appropriate than conventional methods using animal models or traditional cell line assays at each stage because it can reduce the risk to human participants and eliminate the need to use animals while ensuring patient safety and timely delivery of drugs.

### Cardiotoxicity Studies

Drug-induced cardiotoxicity is a major regulatory hurdle in pharmaceutical development. Over 400 medicinal products have been withdrawn from the market since the 1950s, mostly due to cardiovascular (CV), hepatic, and nervous system toxicities (Onakpoya IJ. et al., 2016; Onakpoya I. J. et al., 2016; Kocadal 2018). A systematic analysis indicated that primary cardiotoxicity accounted for 74% of post-market withdrawals, such as arrhythmia (35%), cardiac injury (cardiomyopathy) (38%), and CV events (22%). In addition, during the preclinical to marketing and post-approval stages, CV problems accounted for 22% of withdrawals (Laverty et al., 2011). These data emphasize that the present system of pre-marketing drug safety screening and monitoring lacks sufficient evidence, and that it is necessary to develop adequately specific and sensitive human-compatible models to identify ADRs at all stages of drug development. The standard strategy for *in vitro* preclinical assessment is to inhibit hERG (encoded by *KCNH2*) channels in some heterologous cell types. Despite the reliability of isolating drugs with potential arrhythmogenic properties, the high safety margins of the hERG assay can lead to accurate prediction of arrhythmogenic potential (Gintant 2011). In fact, since the test for the response of the hERG channel and thorough QT/QTc study have been conducted in humans for all drugs before market authorization, arrhythmic reactions caused by QT prolongation have decreased. However, the relationship between hERG inhibition and lethal arrhythmia is not robust. Thus, many safe drugs have resulted in withdrawal from the market (Kramer et al., 2013). To resolve this problem, the Comprehensive *in vitro* Proarrhythmia Assay (CiPA) initiative was established with the intention of advancing safety pharmacology from more traditional pharmacodynamic methodologies toward a combination of in silico and *in vitro* compound toxicity assessments (Gintant 2011; Sager et al., 2014; Yang and Papoian, 2018). The CiPA initiative plans to discover the mechanisms underlying the potential proarrhythmic effects of candidate drugs and is expected to be a safe standard screening tool for drug development (Cavero and Holzgrefe, 2015).

Recently, an international validation study was conducted across multiple sites with 28 blinded compounds, which demonstrated the overall utility of the multielectrode array (MEA) methodology and hiPSC-CMs (Blinova, et al., 2018). They assessed the variation in results among each site, revealing that three predictors, arrhythmia events, delayed repolarization, and repolarization prolongation, can accurately evaluate drug efficacy. These data support that CiPA predicts arrhythmia risk more accurately than the present S7B/E14 guidelines. The Japanese National Institute of Health Sciences (NIHS) also aims to develop and validate a new testing method for more precise prediction of clinical proarrhythmia risk, known as the Japan iPS cardiac safety assessment (JiCSA) (Kanda et al., 2016; Kanda et al., 2018). For example, consortia working with the CiPA initiative and Japanese Consortium for Safety Assessment using the Human iPS Cells initiative developed an *in vitro* proarrhythmia model to test 28 reference compounds with a low, intermediate, or high risk of torsades de pointes using MEA recordings and commercially produced healthy donor hiPSC-CMs (Blinova, et al., 2018; Kitaguchi et al., 2016). These results demonstrated stable reproducibility across multiple sites in categorizing the risk of drugs. Pfeiffer et al. (2016) also developed a kinetic optical recording modality to assess the proarrhythmic effects on Ca^2+^ transients and assessed a larger set of reference compounds. In this study, individual healthy hiPS-CMs within the fields of view were analyzed, thereby increasing the sensitivity of proarrhythmia detection, because it enabled the detection of rare and heterogeneous events such as early afterdepolarizations shown by techniques that examine the whole well, such as MEA. Thus, both CiPA and JiCSA have shown that hiPSC-CMs can be used to evaluate the risk of proarrhythmia and demonstrate the reproducibility and robustness of each assessment system.

Moreover, there are many reports of hiPSC-CM evaluation for drug-induced toxicity, which demonstrate the potential for guiding drug development. Long-term toxicity is a major clinical problem because it can be unpredictable. Even in a well-known drug, calcium channel blockers (CCBs), little is known about drug-specific effects on human CM transcriptomes or physiological alterations after long-term exposure (Lam et al., 2019). Lam et al. simulated the chronic use of four different CCBs and examined both the functional and transcriptomic changes in human CMs, revealing distinct effects on normal CM physiology. Moreover, because the incidence of chronic cancer is increasing, there is an increasing need to address both the short- and long-term cardiotoxic effects of cancer therapies (Gintant et al., 2019). For example, anthracycline doxorubicin (DOX) has both acute and long-term cardiotoxicity. Studies using hiPSC-CMs have shown that continued exposure improves the predictive power for the cardiotoxicity of DOX (Sakamoto et al., 2019). Molecular target drugs, like small-molecule kinase inhibitors, are also related to cardiotoxic effects, such as impaired left ventricular function, myocardial infarction, and arrhythmia (Force et al., 2007). Sharma et al. (2017) screened chemotherapeutic kinase inhibitor drugs and healthy donor lines and observed a good correlation between *in vitro* cardiotoxicity, such as loss of cell viability and contractility, and the clinical incidence of cardiotoxicity. Notably, they demonstrated the cardiotoxicity of VEGFR2/PDGFR-inhibiting TKIs, which act primarily on the cardiac vasculature, showing that cell type-specific toxicities differ between CV and non-CV cell types. The presence of the vasculature in the evaluation of drug-induced cardiotoxicity should be considered, and assays that quantify drug-induced vascular abnormalities should be incorporated and validated using reference compounds (Prigozhina et al., 2011; Vazão et al., 2017). Vascular abnormalities include dysfunction of ECs as well as other cells such as pericytes; therefore, multicellular models containing fluid flow-through vascular networks have been developed, representing a promising option for improving cardiotoxicity tests (Chintalgattu et al., 2013; Savoji et al., 2019).

As described above, electrophysiological abnormalities have been the primary focus of research, although cardiotoxicity is classified into electrophysiological abnormalities, contractile dysfunction, and structural toxicity. The cardiovascular side effects of drugs are commonly caused by CM dysfunction; therefore, contractile function has recently been regarded as an evaluation criterion for the assessment of safety. Saleem et al. (2020) established a multinational consortium comprising four academic teams with two companies and performed a blinded evaluation of 28 drugs with 3D EHTs. They regarded the contraction amplitude as a good predictor of inotropes and demonstrated that the platform-cell accuracy was improved to 93%, which was better than that obtained in *in vivo* animal models. More recently, Rhoden et al. (2021) characterized the impact of sulforaphane treatment, which received regulatory approval for clinical studies targeting neurological disorders and cancer in humans, on CM contractile function using hiPSC-derived EHTs. They demonstrated that acute and chronic sulforaphane exposure reduced CM contraction, perturbed sarcomeric organization, and induced mitochondrial inflation. Thus, hiPSC-derived EHTs have potential of detecting cardiac side effects that have not been indicated in ongoing clinical trials. However, EHTs are generally costly because they require large cell numbers and can only be produced in small numbers. Although maturation status and predictive potential may be inadequate compared to EHTs, scaffold-free microtissues have advantages for large-scale drug screens because they meet the needs of simplicity and smaller cell numbers (2000–5,000 cells/tissue), thereby reducing the costs of each tissue. In the future, miniaturized EHT systems may display the advantages of both systems.

### Clinical Trials in a Dish

An important point in clinical trials is the application of drug candidates to representative samples of the target population to understand the magnitude and distribution of effects that a population at large will experience by taking the drugs. Drug testing with hiPSC-CMs derived from a cohort of patients have been called clinical trials in a dish (CTiDs). CTiDs can overcome safety and efficacy issues, which are the cause of the high attrition rate of drugs in clinical development, by estimating the clinical effects of drug candidates before testing in humans. CTiDs can be conducted for a range of clinical doses at a low cost and outside rigid conventional clinical trial regulations from an earlier stage of the drug development process, whereas animal tests require high costs and extensive resources and their results are subject to interspecies differences. In addition, because hiPSCs can be replicated infinitely, we can generate substantial supplies of identical and inexpensive test materials by using donor tissues. Maintaining an hiPSC bank is a useful strategy for conducting an unlimited number of CTiD studies on an identical cohort of donors. The sensitivity of the tests can be improved by increasing sample sizes. Fermini et al. considered sample size for CTiDs and found that their assay could predict events in 1% of the population with 90% probability using 250 lines (Fermini et al., 2018).

It is also important to consider strategies that incorporate lines carrying rare genetic predispositions to liabilities. For example, some genetic variants can increase the risk of arrhythmia. Accordingly, such lines could increase sensitivity for anticipated events and confirm whether particular genetic variants contraindicate certain drugs. Moreover, CTiDs have the potential to distinguish between responders and non-responders. In practice, Liang et al. demonstrated that healthy and diseased individuals, such as patients with LQT, HCM, and DCM, exhibit differential susceptibility to cisapride, a hERG blocker, and efficiency of CTiDs compared with results for the conventional hERG test (Liang et al., 2013). Similarly, a good correlation between individual susceptibility and drug-induced QT prolongation of moxifloxacin and the hERG blocker sotalol has been recapitulated (Shinozawa et al., 2017; Stillitano et al., 2017). They showed a strong correlation with the susceptibility to QT prolongation on the electrocardiogram and prolonged repolarization of field potential duration in individual hiPSC-CM samples. In addition to arrhythmia evaluation, Burridge et al. recapitulated the clinical susceptibility to DOX using hiPSC-CMs derived from patients with breast cancer who did or did not experience cardiotoxicity (Burridge et al., 2016). They demonstrated that hiPSC-CMs from patients who experienced cardiotoxicity were more sensitive to doxorubicin toxicity with decreased cell viability, impaired mitochondrial and metabolic function, impaired calcium handling, decreased antioxidant pathway activity, and increased ROS production. Kitani et al. also demonstrated that hiPSC-CMs derived from patients who experienced trastuzumab-induced cardiotoxicity had increased cardiac dysfunction (Kitani et al., 2019). These data indicate that CTiDs have the potential to identify susceptibility and predilection. Another contribution of CTiDs is the estimation of adverse drug-drug interactions (DDIs). DDIs are increasing in the aged society and account for 20% of all adverse drug reactions (ADRs) (Reimche et al., 2011). It is important to evaluate safety and efficacy on each patient, a task suited for CTiDs.

However, some challenges of CTiDs with hiPSC-CMs have been reported. A recent study with 16 healthy subjects failed to show a significant correlation between susceptibility to clinical QT prolongation and the APD responses of their hiPSC-CMs treated with the hERG blockers dofetilide and moxifloxacin (Blinova et al., 2019). A potential cause is related to the immaturity of hiPSC-CMs and variation in hiPSC cultures, such as differentiation in batches and hiPSC line variation. Further assessment of more suitable cell lines and culture methods is required to apply CTiDs. Moreover, the drug development process is essential, despite the efficiency of CTiDs. CTiDs are intended to assist, but not replace conventional clinical trials in preventing adverse reactions and attrition earlier.

## Conclusions and Future Directions

HiPSC-CMs induced from both healthy individuals and patients have the potential to recapitulate the molecular and functional features of the human heart. Various efforts have been made to accomplish the functional and morphological maturation of hiPSC-CMs, and many cardiomyopathies have been modeled, improving the elucidation of underlying mechanisms and effective therapies for diseases. Novel drug testing paradigms (CiPA and JiCSA) may be better predictors of the proarrhythmic risk of drug candidates. However, even in 3D fabricated and electrically stimulated hiPSC-CMs, the contraction force was still lower than that in adult CMs, mitochondria were immature, and the sensitivity to Ca^2+^ remained high, which is a limitation of hiPSC-CMs (Hirt, et al., 2014; Ronaldson-Bouchard, et al., 2018). Although various 3D human heart models have advantages and disadvantages, it has been difficult to determine methods to induce the best maturity, because they have not been evaluated in the same way or under the same conditions, and each maturation assessment of 3D tissues lacked precise stratifications (e.g., whole 3D constructs/isolated single CM, or with/without liquid junction potential correction of RMP) (Table 1). The induction of complete CM maturation has yet to be achieved, and patient-specific models of hiPSC-CM have not replaced animal models and traditional cell line assays in new drug development, although 3D models have enabled the recapitulation of disease-specific models that have not been recapitulated in 2D models (Sun et al., 2012; Bliley et al., 2021). It may be necessary to combine approaches mimicking various aspects of the native myocardial microenvironment, including biophysical cues, biochemical cues, coculture with non-CMs, and ECMs. Although the extent to which a more mature phenotype may affect the sensitivity and fidelity of translation of hiPSC-CMs as a disease model remains to be determined, it is important to recapitulate the macrostructure of the heart more precisely. In the future, the robustness and accuracy will be further improved by integrating a greater level of maturation and many readout technologies into new paradigms. Such robustness and accuracy will certainly help in disease modeling, drug efficacy or toxicity testing, and mechanistic studies.

## Glossary

ACM: Arrhythmogenic cardiomyopathy
ADR: adverse drug reaction
AP: action potential
APD: AP duration
ARVC: arrhythmogenic right ventricular cardiomyopathy
BrS: Brugada syndrome
CAD: coronary artery disease
CCB: calcium channel blocker
CF: cardiac fibroblast
CHF: chronic heart failure
CiPA: Comprehensive *in vitro* Proarrhythmia Assay
CM: cardiomyocyte
CMT: cardiac microtissue
CO: cardiac organoid
CPVT: catecholaminergic polymorphic ventricular tachycardia
CSAHi: Consortium for Safety Assessment using Human iPS Cells initiative
CTiDs: clinical trials in a dish
CV: cardiovascular
CVD: cardiovascular disease
Cx43: connexin 43
DCM: dilated cardiomyopathy
DMD: Duchenne muscular dystrophy
EAD: early afterdepolarization
EC: endothelial cells
ECT: engineered cardiac tissue
EHM: engineered human myocardium
EHT: engineered heart tissue
ER: endoplasmic reticulum
ERR: estrogen-related receptor
HCM: hypertrophic cardiomyopathy
hERG: human ether-à-go-go-related gene
hESC: human embryonic stem cell
HF: heart failure
hiPSC: human induced pluripotent stem cell
hiPSC-CM: hiPSC-derived cardiomyocyte
HoC: heart-on-a-chip
hPSC-CM: human pluripotent stem cell-derived cardiomyocyte
IGF1: insulin-like growth factor 1
JiCSA: Japan iPS cardiac safety assessment
LQTS: long QT syndrome
MEA: Multielectrode Array
MI: myocardial infarction
MM: maturation medium
NIHS: National Institute of Health Sciences
NT-proBNP: N-terminal pro B-type natriuretic peptide
PPAR: peroxisome proliferator-activated receptor
RMP: resting membrane potential
ROS: reactive oxygen species
SCD: sudden cardiac death
SQTS: Short QT syndrome
SR: sarcoplasmic reticulum
TdP: torsade de pointes
UPR: unfolded protein response
YAP1: Yes-associated protein 1
2D: two-dimensional
3D: three-dimensional
